# Making sense of mental health in later life: social network dynamics and service access

**DOI:** 10.1080/17482631.2025.2581516

**Published:** 2025-11-08

**Authors:** Dominique Jana, Christoph Heuser

**Affiliations:** Department of Gerontology, Centre for Research on Ageing, University of Southampton, Southampton, England

**Keywords:** Mental health, social networks, barriers and facilitators, mental health access, later life, reflexive thematic analysis

## Abstract

**Purpose:**

Using a qualitative approach, this study explores how older people have experienced barriers and facilitators in access to mental health services (MHS). It uses the life-course perspective and the Network Episode Model (NEM) as theoretical frameworks. It addresses three research questions: 1) How have older people experienced access to MHS as older adults and at other times during their life-course? 2) How and why do older people decide to access MHS? and 3) What barriers and facilitators have older people experienced when wanting to use MHS?

**Methods:**

Four in-depth semi-structured interviews were conducted with older people (65 +) using mental health services, which were recruited using purposive sampling. The results were analysed using reflexive thematic analysis, considering the subjectivity and reflexivity of the researchers.

**Results:**

Three themes were identified as the main processes where barriers and facilitators are experienced: 1) Recognising that there is a mental health problem and you can get help, 2) Destigmatisation of mental health, and 3) Need for integral support

**Conclusions:**

Participants’ past experiences and knowledge of mental health seem to contribute to help-seeking. Health and mental health professionals, especially GPs, can facilitate access to MHS by recognising, validating and integrating older people’s needs. Conversations about mental health with personal and community organisations can lessen stigma and increase social support. Policies should continue promoting mental health literacy and awareness, and adopt a holistic delivery model from a gerontological perspective that includes older people and their social networks.

## Introduction

When referring to access to mental health services (MHS), a broad literature shows that older people are the age group with the lowest rate of access to these services (Cairney et al., 2010; Garrido et al., 2011; National Health Service, 2015; O’Donnell et al., 2021; Sharland et al., 2023; Volkert et al., 2018). In the United Kingdom (UK) studies have also shown that older people have the least access to the National Health Service (NHS) Talking Therapies (previously known as Improving Access to Psychological Therapies (IAPT)), a program from the National Health Service (NHS) that offers evidence-based talking therapies for outpatients with Common Mental Health Disorders (CMDs) (National Health Service, 2015; n.d.; Pettit et al., 2017; Sharland et al., 2023). In the UK, mental health services for older adults are delivered primarily through the National Health Service (NHS), which provides free services at the point of access. General practitioners (GPs) act as the first point of contact. These services include community mental health teams and inpatient units for complex cases. In addition, people with mental health problems can self-refer to specific services. However, despite this infrastructure, older adults consistently show the lowest rates of access to mental health care, which highlights the importance of understanding the barriers they face.

Most studies on access to MHS have used quantitative or mixed methods to understand access to mental health in older people, whereas qualitative methods have been used less frequently. Quantitative studies have the strength to generalize the results to the population, and to explain the effects of several factors on this access (Clark et al., 2021), however, they have the limitation of understanding mental health as a restrained and static experience. From a positivist epistemology, this concept is limited to symptoms, disorders, and specific moments of access.

In the quantitative literature, several factors have been explored. Between them, the only consistent factor is the perceived need for help, suggesting that older people perceive less the need to access these services (Garrido et al., 2009; Mackenzie, Pagura and Sareen, 2010; Forbes et al., 2017; Rens et al., 2020; Mackenzie and Pankratz, 2022). Other factors that have been emphasized are mental health literacy (Hannaford, Shaw and Walker, 2019; Knight and Winterbotham, 2020; Mackenzie and Pankratz, 2022) and the stigma attached to having a mental health problem and access to services (Hannaford et al., 2019; Knight & Winterbotham, 2020; Lavingia et al., 2020; Mackenzie & Pankratz, 2022; Muir-Cochrane et al., 2014; Rens et al., 2020). Among systemic factors, costs and distance transportation have evidence of influencing access (Forbes et al., 2017; Knight & Winterbotham, 2020; Lavingia et al., 2020; Muir-Cochrane et al., 2014; Pepin et al., 2009). Additionally, the role of General Practitioner (GP) (Hannaford et al., 2019; Knight & Winterbotham, 2020; Lavingia et al., 2020; Pettit et al., 2017; Walters et al., 2018) and social support appear to influence how health access is managed (Bretherton, 2022). These factors allow us to better understand access in older people; however, the lack of consistency on their effects makes us believe that it is relevant to explore how these factors interact in the dynamics of accessing MHS, as they could not explain access on their own.

Regarding the restricted conceptions of mental health of quantitative studies, most of them use the dominant models of service use (Andersen’s behavioral model, health belief model and theory of reasoned action), which consider that the individual makes a rational, voluntary, and active choice to meet their health needs (Pescosolido & Boyer, 2009). In these studies, mental health is understood as symptoms, and access is limited to static moments. By using qualitative methods to understand access to mental health, these limitations can be mitigated. These can be useful to comprehend the complexity and richness of older people’s experience of accessing health services (Braun & Clarke, 2013). They can contribute to having a more profound and thick understanding of access and capture the experiences and the subjectivity of older people (Braun & Clarke, 2013).

A framework that is useful for expanding knowledge about access to MHS in older people is the Network Episode Model (NEM). Contrary to dominant models of service use, it understands mental health problems as a social process managed through social networks, the treatment system and social service agencies (Pescosolido, 2011; Pescosolido & Boyer, 2009). In the NEM, the individual is a pragmatic user, and their decision to use services is not always a result of cost-benefit calculus but also of the interaction with others who may recognize or deny the problem. Rather than a “yes-no one-time decision”, this considers pathways of practices during an “episode of illness” where power, structure and content of social networks must be understood, being these pathways part of an “illness career” (Pescosolido & Boyer, 2009, p.436).

In this model, social networks provide the structural element of social interaction, the latter being the underlying mechanism “at work in the recognition, diagnosis and treatment of mental health problems” (Pescosolido, 2011, p.16). These pathways are understood in the interaction of social networks at different levels and times. The NEM has the strength of considering this process as dynamic and gives the same weight to individual and social factors, focusing on the interaction between these and the different networks. It also has the strength of considering different pathways in the illness career of a person, where the interactions and outcomes can vary or be similar across different pathways.

Two studies have used this framework within a qualitative approach, to capture the access complexity. Berard et al. (2020) show that most older people (60 +) who accessed outpatient mental health treatment from clinical psychology experienced more than one pathway to treatment, where the majority started by feeling “not well” and then they were bounced in helpful and unhelpful ways through the formal and informal system until they found adequate treatment (p.5). According to their results, most people chose to access or did not actively seek help, but did not resist it. The researchers believe this could mean that participants are willing to access treatment and, at the same time, are not in charge of it. In this process, older people may not know how to navigate the system, and the system is not supportive. Beatie et al. (2022) also used the NEM as its theoretical framework. By analyzing interviews with older people from a geropsychological clinic, they concluded that this framework could capture the complexity of these experiences. Their results contribute to the understanding that what influences older people’s pathways in seeking help is the support of social networks, the referral system (adequate or inadequate referrals), and the knowledge of treatment options. Both studies have the strength of understanding access as a dynamic process where the older person interacts with their social networks.

From a gerontological perspective, the life-course perspective can also be useful to understand how older people have accessed MHS over time. This perspective allows an understanding of individual experiences and the influence of historical, cultural and social factors over time (Bengtson et al., 1997). In this perspective, humans build their worlds through co-constructed relationships organized by practices and social institutions, affected by social and cultural meanings (Dannefer & Phillipson, 2013). These institutional contexts “define the normative pathways of social roles, including key transitions, and the psychological, behavioral, and health-related trajectories of the person as they move through them” (Bengtson et al., 2005, p.493). This perspective can be coherent with qualitative methods, as the focus is on individual processes and experiences considering social factors.

The gap in the literature is that quantitative studies are not sufficient for the understanding of access to mental health in older people, as results on the effects of factors have not been conclusive. This emphasizes the need for more qualitative studies that explore the complexity of the dynamics of older people accessing these services.

### **Present study**

This study follows an exploratory qualitative approach to explore the different meanings attributed to the experience(s) of accessing MHS, placing the participant’s subjectivity in the center (Braun & Clarke, 2013). It uses the NEM and the life-course approach as its frameworks. It aims to explore how older people have experienced barriers and facilitators in access to mental health services in Southampton, UK.

## Methods

This study explored how older people have experienced barriers and facilitators in access to Mental Health Services (MHS). It addresses three research questions: 1) How have older people experienced access to MHS as older adults and at other times during their life-course? 2) How and why do older people decide to access MHS? and 3) What barriers and facilitators have older people experienced when wanting to use MHS? Examples of the questions used in the interviews were “What motivated you to seek access to mental health?”, “Can you tell me about your experience with the mental health service the first time you visited it?”, and “In what ways do you think it is similar or different to go to a mental health service as an older adult than going at other stages of life?”.

An exploratory design within a qualitative approach has been chosen, which has the strength of accessing the subjective experiences of older people who have used these services. This allowed getting to know the participants’ meanings and experiences, understanding their mental health and access conceptions, and how they make sense of this process (Braun & Clarke, 2013). Using a qualitative approach also contributes to considering the particular context in which the participants and the researchers are situated, reflecting on the different systems of values and cultures that could exist within the different social interactions. Furthermore, the research used constructionism as its epistemology, which considers that knowledge about the world is constructed through systems of meaning and shared communication (Braun & Clarke, 2013). Relativism ontology underpins this epistemology, where there is no singular underlying reality but different realities specific to social and cultural contexts (Braun & Clarke, 2013; Hammersley, 2013). This enables understanding the meanings that older people attribute to their experiences in accessing these services by understanding how their interactions with others create different realities (Flick, 2023). It is also relevant to consider the researcher’s subjectivity by being analytic and reflective (Lumsden, 2012). In this case, DJH was aware of the characteristics that influenced her role, such as her background as a psychologist, her Latin nationality, and English as her second language.

Four in-depth semi-structured interviews were conducted with older people aged 65 and over who were using mental health services as outpatients. Despite extensive recruitment efforts through social, community, and religious organizations, as well as outreach to mental health organizations, only four participants were ultimately recruited. Recruitment was therefore more limited than anticipated, reflecting organizational constraints and the possible stigma associated with discussing mental health in later life. Although the sample is small, qualitative research emphasizes depth and richness over breadth. Reflections on each interview indicated that participants provided sufficiently detailed accounts to enable a nuanced exploration of barriers to accessing services. Recruitment challenges are discussed further in the Limitations section.

Each interview lasted around 40 minutes and was conducted by DJH. The use of a semi-structured interview allowed the creation of meanings and understanding of older people’s experiences in the interaction between the interviewer and the interviewees (Edwards & Holland, 2013). As conducting interviews involves a dynamic of power and emotions, DJH had to be aware of her position as a researcher and the feelings that appeared during the interview *(ibid.)*. For this, a reflexive diary was used to reflect on different emotions experienced through the different encounters. Two interviews were conducted online, one by phone and one face-to-face in the participant’s home. They were available, convenient, accessible, and private options for both, the researcher and the participants (Edwards & Holland, 2013). Using different forms of interviews allowed to include older people with different characteristics and needs (for example, people who lived far away, people with no internet, and people who could not go out of their homes because of disabilities). All of these forms of interviewing allowed the researchers to access older people's experiences and meanings by building a relationship based on trust (Braun & Clarke, 2013).

All participants were people aged 65 years and over who were using a mental health service during their later life. One man and three women participated, all White British. The reasons they used MHS varied across the participants, ranging from the need for support to common and severe mental health disorders. Their pathways to treatment varied: three participants had mental health conditions throughout their lives and received different treatments, while one participant began receiving treatment later in life, referring to having had a mental health problem for a long time. They all accessed MHS by using the National Health Service (NHS), complementing this, in some cases, with private providers. The following table briefly describes their pathways to mental health care (see Table 1).

**Table 1.** Brief description of pathways to mental health care.

Pseudonyms	Age	Brief description of pathways to mental health care
Andrew	70	Andrew lives alone in a flat and has been living for 53 years with mental health conditions. He has been diagnosed with depression, schizophrenia, and obsessive-compulsive disorder (OCD). His mental health conditions started after he was sexually assaulted. Throughout his life, he has been accessing different treatments, such as medication, depots and electro-treatment, and seeing different professionals, such as doctors, nurses and psychiatrists. He sees his GP and a well-being nurse from his health center for mental health. They ring him or visit him. For treatment, he receives medication. Andrew cannot go out of his flat because of his OCD. He feels supported by his sister and nephew but not by his neighbors.
Emily	73	Emily is going to counseling for post-traumatic stress disorder (PTSD) at a public mental health service. She mentioned that she had different traumas across her life, but last year was the first time she received treatment for this. The Pain Clinic referred her. Her GP gave her antidepressants but did not refer her to a mental health service. Emily is almost in the middle of her treatment; she is receiving Eye Movement Desensitization and Reprocessing (EMDR), the recommended therapy for trauma.
Eleanor	65	Eleanor lives alone in a flat. She has many friends and is very active in her community. She is currently going to a mental health service and waiting to access another one. She is going to MHS because she has been having anxiety, depression, and panic attacks. She first started using MHS a long time ago; she does not remember when. The first time she accessed it, she started going to a community association. Now, she is going to a private provider and waiting to go to a new mental health service, which is public. The first time she accessed MHS, her GP referred her. She has now self-referred to the mental health provider and is waiting for attention.
Adeline	74	Adeline lives alone. Her family and friends live in a different city, and her husband lives in a care home in the same area. Her husband has dementia and is receiving end-of-life care. She started receiving mental health support from a public mental health provider as she was going through different situations that made her feel she needed support. She received this through phone calls as she could not attend the service because of her disability. Adeline used to work as a nurse and has experience doing counseling for grief and bereavement. After three sessions with this provider, they discharged her because they determined she had problematic consumption of alcohol. They referred her to an alcohol and drug center, and she is waiting for attention. The first time Adeline went to a mental health service was around 48 years ago, where she voluntary checked-in in a psychiatric clinic after a crisis.

Interviews were transcribed and anonymised by removing any possible identifiers. Transcripts were analyzed using reflexive thematic analysis (RTA) with the software NVivo version 1.7.1. This allowed the researchers to develop, analyze, and interpret patterns across the dataset, creating codes that developed into themes (Braun & Clarke, 2022). RTA is a method among the family of thematic analysis, which follows a full qualitative approach. According to its epistemology, the process of analysis is interpretative and subjective. It is centered on the researchers’ subjectivity, deep reflection, and engagement with the data (Braun & Clarke, 2019). This allowed the researchers to consider socially constructed meanings and experiences and consider participants’ interactions with their social networks. The analytical process followed the six phases proposed by Braun and Clarke (2022). The first phase was familiarization, where interviews were listened to again and re-read. In this process, notes about insights or ideas were taken within the interviews and the whole dataset. The second phase was coding, where initial code labels were defined, evolving to final codes by reading and coding the different interviews several times. The coding was inductive and semantic, but also deductive and latent. In the third phase, for generating initial themes, codes were clustered concerning tentatively core ideas. Some of the codes were re-labeled and re-grouped in this process. After developing candidate themes, in phase four, these were reviewed considering the whole dataset. Some were merged, and others were renamed to describe the core concepts better. In this phase, the relationship between the themes and how they answered the research questions was considered. Consecutively, in phase five, themes were defined and given their final name, ensuring that they were built around a singular central ideal, illustrated richness and diversity, were not too multi-layered, and had clear boundaries. Finally, the results were written descriptively in phase six, including the interpretative element in the discussion (Braun & Clarke, 2022). This research has ethical approval from the University of Southampton.

Finally, to ensure the trustworthiness of the data, several strategies were employed. A semi-structured interview guide was used to promote consistency across interviews while allowing flexibility for participants to elaborate on their experiences. All interviews were transcribed verbatim, and transcripts were read and re-read to ensure familiarity with the data. Thematic analysis was conducted systematically, with codes and themes refined through iterative discussion between DJH and CH. A coding tree and clear code definitions were developed to guide coding decisions, and reflexive notes were maintained throughout the process to enhance transparency and confirmability.

## Results

In total, three themes were identified: 1) Recognizing that there is a mental health problem, and you can get help, 2) Destigmatisation of mental health, and 3) Need for integral support (see Table 2). These themes were developed considering the different experiences across the life course, where barriers and facilitators were encountered within these processes.

**Table 2.** Themes summary.

Theme	Characteristics
Recognizing that there is a mental health problem, and you can get help	This theme refers to the process of the person making sense that what they are experiencing is related to mental health. In this process, the treatment system, especially GPs, have a relevant role in helping to understand what is happening to the person and to offer treatment. This includes also knowing that help is available and can be reached. Also, the types of available treatments. In this process, the person is willing to get better.
Destigmatisation of mental health	This theme refers to the stigma of mental health changing over time, where there is more awareness about it and more openness to talk about it, making it easier to share personal experiences related to mental health. Destigmatising mental health relates to the idea of understanding mental health as something that everybody experience. In this theme there is a movement over time from experiencing more stigmatization to less stigmatization.
Need for integral support	This theme refers to the need of older people on receiving support based on all types of needs (biopsychosocial) and not just on physiological needs. This includes having a comprehensive view of mental health problems, validating, and understanding the resources and context of the person. Also, having a close and warm relationship with professionals but also with people from their context, which reflects the need of being socially connected through treatment but also as an outcome of treatment. Whether if the person receives integral support or not is related to adequate access to MHS. When the person does not receive integral support based on their needs, they can be bounced within the system in helpful and unhelpful ways.

### **Recognizing that there is a mental health problem, and you can get help**

The first theme reflects that participants recognized that they have a mental health problem and that there is help. Participants mentioned that when they accessed MHS for the first time, it was through their GPs. In this process, before going to their GP, they felt that something was not right. The health system seems to be crucial in helping them make sense of what they were experiencing. Health professionals were the ones who helped them understand that their feelings and experiences were related to their mental health and that they could get help for that. In this process of accessing for the first time, the health system enabled access to treatment by offering different treatments at different times (or not). For example, Andrew went to his GP after being sexually assaulted, where his GP helped him make sense of his experience and offered him treatment. On the other hand, if they are not helped to make sense of what they are going through, they may not recognize the need for treatment and consequently miss out on access to MHS. Emily expresses,

*“I have meant, I couldn’t even think that I had mental health problems. It didn't even occur to me. But as I said, when Southampton Pain Clinic referred me to SW [mental health NHS provider] and I spoke to them, they did say “You've got PTSD” and I said, “Well, I do know that”. They said, “Well, it's a mental health problem”. I don't know why I didn't connect the two. I'm not stupid, but I just didn't connect the two being PTSD a mental health problem. I thought it was more emotional problem” (Emily).*

Making sense of experiencing a mental health problem is also related to knowing the available treatments and how they can help. In this sense, there is a need for advertising more MHS so people can know where and how to receive help.

For people who have accessed MHS more than once, their past experiences and knowledge about mental health have helped them make sense of what they were going through and, therefore, ask for help. Eleanor says,

*“I'm also waiting for counseling which is better than what I was, with SW [mental health NHS provider] cause that's free. […] That's free and you can refer yourself. So, I've, so I've referred myself. After talking to Christine [a friend] and then to unions and counseling, but I'm better than what I was" (Eleanor).*

In all these experiences, there is a need and a willingness to sort out problems related to mental health and feel and be better. Even when the treatment system does not respond to people’s needs, they keep trying to get help. Adeline, who was discharged after her three sessions of therapy, says,

*"So no, I'm really quite disappointed, I think. Um, I think I do. I could do a better job myself, to be honest with you [Laughs] You know, I'm sorry, I don't mean to be, to sound arrogant or anything, but I'VE GOTTA GET BACK. I'VE GOTTA GET BACK IN THE SADDLE. I REALLY HAVE. Cause I really want to help people as well. You know, with, with my experiences as well. Really" (Adeline).*

The three other participants also wanted their situation to change and improve. Wanting to change their situation was crucial in wanting to access MHS. In this scenario, whether the health system responded or not to this need, acted as a facilitator or a barrier in the use (realized access) of MHS.

#### **Destigmatisation of mental health**

The second theme is about the destigmatisation of mental health that the participants experience. The study shows that perceiving less stigma around mental health can also contribute to being more open about personal mental health experiences and asking for help. Destigmatising mental health happens by learning about mental health problems from others while simultaneously benefiting others by sharing experiences. As Emily expresses, her daughter’s experience with mental health problems allowed her to feel less stigmatized, but also empowered her to share her own experience with people from her church,

*“I think so, yes. As I said, I think that I think our attitude and mental health is just, you know, people who are insane or, you know, or suicidal. But I've just realized there's, there's many different areas of mental health. So that I've learned, and now I'm not ashamed of, like, and I said a few people at church going to counseling. Obviously, I don't tell them why I'm going to counseling, but I don't feel ashamed of going counseling because I think people got to know that you have traumas and problems and, and people need to understand that sometimes you need help. Through him, and I'd just tell people I'm going to counseling and that's it, yeah. And I think that, that, I mean my daughter's got very bad mental health issues. And I think in the last 10 years that the stigma of that has lessened quite a bit, so I don't feel anything wrong and going there, I know that it's such a wide area that they offer” (Emily)*

Destigmatising mental health also refers to the idea that everyone can experience mental health problems; therefore, sharing these mental health-related experiences can allow others to be more open about mental health and feel less isolated in the experience.

*"So, I don't have any, I don't have any problems telling anyone if I have to tell them all, it just comes out naturally. I well, you know, “I have had mental health problems or I'm going through this”. And people are very open then about how, how they, what they go through, which almost gives them permission to say, “Well, actually, you know, I've, I've been going through the same Adeline”, and I think, well that's rather nice to be able to share these experiences, isn't it?" (Adeline)*

Participants also mentioned that people are talking more about mental health in different media, especially television, which creates more awareness about mental health and, therefore, lessens the stigma. In this process, one of the participants emphasized that it is important to create awareness by considering all ages. She says,

“*They're using their platform to talk about mental health and it's SO important, isn't it? It doesn't matter how old you are, you know so"* (Adeline)

In destigmatising mental health problems, participants recognize that when accessing Mental Health Services (MHS), sharing mental health experiences is relevant as it allows them to feel less ashamed and open about mental health, allowing others to feel the same way.

#### **Need for integral support**

The final theme concerns the need for integral support in the healthcare sector. Having an integral support means being able to respond to different types of needs, such as social, psychological, and biological needs, articulating different treatments and services. Also, having a comprehensive perspective recognizing the person’s experience and resources over its diagnosis.

The different participants' experiences reflect that when a mental health problem is recognized and addressed in all its dimensions, the person has a better chance of receiving adequate treatment. However, for individuals experiencing multiple mental health problems, receiving adequate treatment becomes complex. The treatment system might 'bounce them around,' offering different and isolated treatments that do not necessarily complement each other. An example of the former is the experience of Emily, where she receives Eye Movement Desensitization Reprocessing (EMDR) for her Post-Traumatic Stress Disorder,

*“When I first went to the first counseling, they gave me a choice of being treated by initials, CBT [Cognitive Behavioral Therapy] or EMDRI, I don’t' know what the initials are. But anyway, I dealt with my counselor, and EMDRI was the best way for me. It was more direct, so we've, I've been doing about six or seven sessions now. And it has helped in two areas. Yes, so it's, it's helping. I've got another I think 12 or 16 sessions to go, but it, it has helped so far. Heavy stuff is yet to come. So, I'm not looking forward to, but it's got to be done”* (Emily)

In the process of accessing MHS, in the participants’ experiences, the health system, especially primary care, was more focused on their physical/biological needs, responding mainly with medication towards mental health problems. About this, participants refer that receiving medication is not enough, expressing other needs (psychological and social) that are not addressed. Andrew, who is living with schizophrenia, is only receiving medication as treatment. He said,

*“No, I don't, I don't. I don't think they're doing enough. Cause I’d like to be able to go out and say to my neighbors, “I’m very sorry to hear me talk to myself and saying silly things, please forgive me”, you know. “PLEASE TALK TO ME”” (Andrew)*

This reflects that accessing MHS also means addressing mental health needs comprehensively rather than just focusing on the symptoms. Emily expressed that as a first option, she was put on medication, which did not address her problem as her suffering was not acknowledged,

*"Maybe I just. I don’t know. I could have just said that was something's happening. I don't know. I can't cope. I don't know what I was said, but whatever it was they put me on the tablets. This is, this is not the latest trauma. It's one of the traumas going back a few years. But I can't ever remember discussing anything to do with what I was suffering; about lot of the traumas I've suffered" (Emily).*

This is related to the need to receive humanized attention, which means being understood and validated by health and mental health professionals. Humanizing attention involves considering the person’s resources and context. When this does not occur, frustration appears as the mental health need persists over time. Sometimes, this can lead to looking for solutions outside the treatment system.

*"And Dominique [interviewer], we're not robots. We're human beings. And when somebody comes along and starts to do it using a tick list and the voice is monotone. Tick, tick [makes the noises of ticking boxes] That does not bode well with me, Dominique [laughs], I’m sorry, it doesn't. Doesn't bode well with me at all" (Adeline)*

On the other hand, one of the participants expressed being validated in her needs, which emphasized the importance of this element in the utilization of these services.

*"Well, because well SM [mental health charity]. Don't know if you know, but most of the staff have had some sort of mental health problem or got it. So obviously they understand a bit more than what some people would, you know." (Eleanor)*

Another integral support element is the response to the need to be socially connected, which is expected to be achieved through treatment. Participants expressed this by considering social connection as a relevant reason for going to a mental health service, as significant for going through treatment, and as a main outcome of treatment. Wanting to be socially close to others can be one of the main motivations to go through treatment, and the health and mental health treatment responding to this need can enable the use of these services.

Participants referred, explicitly or implicitly, to wanting to be socially connected as one of the main reasons for accessing MHS. For example, Emily expresses that social connections are important to her and that she can see that in her improvements,

*"Yes, yeah. But as I said, there's a couple of things that I wouldn't have done before the treatment that I was able to do. Ermm, socially and social, social gathering. And so, I thought, well, I was quite pleased with myself when I managed to do it, I thought I never have done that before, so something must be working" (Emily)*

For this reason, it is essential for professionals to be empathic and approachable to the participants. In the case of participants who have disabilities and cannot go to the service, alternative delivery options, like phone calls and visits, sometimes lack this emotional closeness from professionals. Andrew expresses,

*"Well, Amy [his carer] could phone me at least once a week until she comes to visit. But doctors, when you phone them up now it's awkward to get hold of them." (Andrew)*

Feeling socially connected with people from their personal network is expressed as relevant for going through treatment. This can be achieved by support groups, family members or friends. Eleanor mentions the importance of one of her friends in her process,

*"Mmm. Oh gosh. Well. I used to go to this house sometimes and we were in a group then, you know, lots of people were talking about things. But I am very lucky because I have got quite a lot of people that I can talk to, and my friend Jane. She's a guiding friend, but she's a very nice lady. She's so 70 and she's also got a mental health qualification. So, we go out sometimes to the theater or something like that and obviously we, she will understand you a little bit […] because she's got this all-mental health qualification, if you know what I mean? I get quite a lot of help through her guiding" (Eleanor)*

This makes it less difficult to go through treatment and makes experiencing mental health problems easier.

## Discussion and conclusions

Exploring the experiences of older people who have used MHS in Southampton allowed the researchers to understand participants’ pathways to treatment by recognizing constant processes across different stages of life and elements that become more relevant in later life. The life-course perspective contributed to understanding individual experiences and how the previous ones influence the latest experiences. At the same time, it allowed for the analysis of the influence of social contexts and institutions over time, especially the treatment system (Bengtson et al., 2005). This perspective aligns with the NEM, which emphasizes the influence of social networks on access to MHS. Using this model contributed to understanding the complexity and dynamism of accessing MHS by focusing on the different processes that can occur through the life-course as part of an illness career (Pescosolido, 2011).

In the experiences of the participants from Southampton, adequate access depended mainly on the health system's response in a context where participants were willing to solve their mental health problems. Specifically, GPs had a pivotal role, consistent with being a source of advice and referral (Hannaford et al., 2019) and the preferred source of access (Lavingia et al., 2020). The opportunity for GPs to respond was given in a context where participants wanted to feel better, not necessarily knowing that they were experiencing a mental health problem. This can be related to older people perceiving less of a need to seek professional help for their mental health (Garrido et al., 2009; Mackenzie et al., 2010; Forbes et al., 2017; Rens et al., 2020; Mackenzie and Pankratz, 2022). In this age group, they might perceive that they need help and feel unwell, without necessarily linking these feelings to a mental health problem. This idea is consistent with the results of Berard et al. (2020), which show that when older people were seeking help, they started feeling not well, being unsure of what to do and being bounced by the system in unhelpful and helpful ways until they found adequate treatment.

Considering the life-course perspective, GPs were more relevant the first-time participants accessed MHS, as they helped them recognize their mental health problem(s) and offered them treatment and referral. In this context, it was relevant not just to give them a diagnosis but to help them understand what they were experiencing. In this process of making sense, knowing the available treatment options and how they work facilitated access, consistent with Beatie et al. (2022) results showing that a lack of information about treatments can delay seeking psychological treatment.

Participants’ previous experiences and knowledge about mental health also contributed to seeking MHS during the subsequent episodes of illness (Pescosolido & Boyer, 2009). This can be related to mental health literacy, described as a strong predictor in older people accessing MHS (Mackenzie & Pankratz, 2022). According to Jorm (2000), mental health literacy consists of the ability to recognize mental health problems or psychological distress, the knowledge about causes and risk factors, available interventions and how to seek mental health information, and attitudes that facilitate the recognition. In participants’ experiences, mental health literacy seemed to facilitate access during their next episodes, where it was relevant not just to know about mental health but to be able to articulate this within their own experiences. This emphasizes the role of social networks in helping to make sense, consistent with the results from Reynolds et al. (2022), where older people preferred to obtain information by discussing it with family members, friends and healthcare providers.

In recognizing a mental health problem and knowing that treatments can be helpful, the association between the steps in previous pathways to treatment and the outcomes seem also to affect future experiences of access. This means that if in previous experiences the person went to a health provider, understood what they were going through, and then had positive outcomes in treatment, this association of a positive experience can contribute to their willingness to access mental health services again. This relates to using more MHS if one has a history of mental health or a previous-year mental health disorder (Garrido et al., 2009, 2011), where it may be important not just to have previous experiences but also to have positive previous experiences. This emphasizes the relevance of improving mental health delivery services throughout the life-course, as people may learn from these associations.

While accessing MHS, stigmatization also appears to be relevant. Perceiving less stigma around mental health allowed participants to be more open to talking about what they were going through. Stigma can be related to mental health literacy, as talking more about mental health can contribute to a deeper understanding of what this means (Simões de Almeida et al., 2023). At the same time, participants perceived that public stigma has lessened, consistent with previous studies (Hannaford et al., 2019; Knight & Winterbotham, 2020).

In the process of destigmatising mental health, another relevant idea identified is that everyone can experience mental health problems. This can be related to *common humanity*, one of the components of self-compassion, defined as the recognition “that all humans are connected, that we all fail, that we make mistakes and engage in dysfunctional behavior” (Wong et al., 2019, p.418). These authors found that this may buffer the effects of public stigma on self-stigma. In future research, the role of self-compassion (and compassion) could be further explored, as it seems to facilitate access to MHS. For participants, common humanity was experienced in the interaction with personal and community networks, specifically family, friends, and church, emphasizing the importance of being able to discuss mental health in these contexts. Also, experiencing this could be related to social inclusion, as when participants experienced common humanity, they felt less ashamed and more supported by their networks.

Finally, the need for integral support has been discussed in the literature as the need to use integrated or holistic models of care in older people, which are models that are “comprehensive, multidisciplinary, accessible and integrated with other health and social services” (p.220) and are based in the belief that mental health problems are caused in the interaction of psychological, physiological and social factors (Biering, 2019). This model has been described as the most helpful for older people, as the focus is not just on symptoms but on the development of new meanings and purpose, considering the adaptation to age-related changes (*ibid.*)

Receiving integral support (or not) seems to influence older people’s pathways to treatment. The experiences of the participants show that if they receive treatment based on their needs, they will have positive outcomes, contributing to increasing their use of MHS. However, if the person receives different treatments that are not based on their needs, they will probably be bounced within the system, accessing different services that do not always respond to their needs. This is related to participants’ receiving treatment focused on medication and physiological needs rather than an integral intervention that considers psychological, social and physiological needs. Most of the time, receiving attention based on the medical-psychiatric model was felt as insufficient. As a consequence, their mental health needs persisted over time, leading to access to the health system several times for the same reason. This is important to consider as the literature shows that medication is the most common intervention for older people who experience mental health problems (Biering, 2019). On the contrary, research shows a preference of older people for non-pharmacological treatment over medication (Lavingia et al., 2020).

Considering older people’s needs is also related to considering the resources and context of the person, validating their experience. McCormack and Skatvedt (2016) refer to this as a need for recognition and respect for older people’s perspectives, emphasizing the need for a person-centered approach which focuses on the collaboration between the “service users” and the “care providers” (p.104). Their results showed that when the attention is task-oriented, this provides little opportunity to emotionally engage, creating the impression that health workers do not care about older people’s experiences. This is consistent with the participants’ experiences, where this disengagement was more pronounced when they did not have the opportunity to attend the services, receiving just phone calls or visits. Validating older people’s experiences becomes important as older people could require higher energy levels to express and meet their needs *(ibid.).* Additionally, research has shown that transportation can be a barrier to older people who have difficulties traveling to these services (Muir-Cochrane et al., 2014; Pepin et al., 2009). In this research, not being able to travel to the services was only a problem when the other delivery options did not allow for social engagement and validation of older people’s experiences. This is relevant as alternative delivery options could be a suitable option for increasing access for older people with transportation difficulties, such as people with disabilities or remote locations if they adopt a person-centered approach.

Another relevant aspect of integral support is the need to be socially connected with health and mental health professionals and people from their personal and community-based networks. Regarding the reasons for going to a mental health service, needing to be socially connected was one of the most important reasons for the participants. This could be related to the presence of loneliness being associated with higher mental health needs and service utilization (Bao et al., 2021). However, this could also be related to potentially feeling lonely, as some of the participants with social support referred to feeling more distant from the people they cared for, mentioning, in some cases, to expect to be more socially connected as an outcome. Furthermore, the literature has shown that social support has a complex role in service use, as having more social support can reduce access to MHS and lessen the psychological distress being addressed outside the treatment system (Thoits, 2011). At the same time, research shows that if help-seeking intentions accompany social support, it can increase the use of MHS (Bretherton, 2022). Whether social support mediates access or not is not addressed within this research. However, these results show that for older people, it is relevant to be socially connected and feel socially supported as an outcome of treatment and for going through this process. This is consistent with Bjørlykhaug et al. (2022), who conclude that social support is crucial for preventing mental health problems, maintaining good mental health, and facilitating recovery.

Participants also referred that social support was relevant for them to go through treatment, allowing them to go through a difficult process accompanied but also contributing to help them in their recovery. The relevance of the social component of mental health in older people emphasizes the need to deliver an integrated response and the importance of strengthening social inclusion within personal and community-based networks (Bjørlykhaug et al., 2022). This could help respond to mental health needs at different levels and increase the use of MHS by having more positive outcomes.

A qualitative approach contributed to comprehending the influence of social networks on participants’ understanding of mental health and their decisions in accessing MHS (Pescosolido & Boyer, 2009). Using in-depth interviews allowed us to understand different experiences in access to MHS by having the possibility of exploring each one of the different experiences through the life-course. This topic was sensitive to the participants, and it was relevant to establish horizontal and respectful relationships with them (Dickson-Swift, James, and Liamputtong, 2008). Offering flexibility on the type of interview allowed participants to have control over the dynamic of power, as interviews can be asymmetrical as the interviewer has the power to ask questions (Braun & Clarke, 2013; Edwards & Holland, 2013). Qualitative methods then, can contribute to analyzing the complexity of access in mental health by establishing horizontal relationships, being particularly relevant in a context where older people may feel powerless about their mental health.

## **Conclusions and Policy Implications**

Barriers and facilitators in access to MHS are experienced by these participants in the interaction between them and their social networks, especially GPs. The recognition of mental health problems and the knowledge about treatments available and their effectiveness, the presence of stigma, and how the system responds to different needs appear to influence their different pathways to treatment. These processes seem relevant in various stages of participant’s lives, emphasizing the need to promote mental health literacy, reduce stigma, and move towards a holistic approach to address mental health throughout their life-course. Moreover, in the experiences of access in later life, it seems that age-related changes are not necessarily considered by the social networks, emphasizing the need to promote gerontological knowledge as well, understanding the importance of integrating social, psychological, and physiological needs, and responding to them in a respectful and validating way. This highlights the relevance of the social component of access to MHS in older people, where the lack of a comprehensive understanding of older people’s mental health could contribute to social exclusion.

Mental health literacy should be promoted to increase access, consistent with the policy recommendations from other studies about access to MHS in older people (Berard et al., 2020; Forbes et al., 2017; Knight & Winterbotham, 2020; Mackenzie & Pankratz, 2022; Volkert et al., 2018). Mental health literacy has a relevant social component as it seems to be acquired in the interaction with personal, community-based and organizational networks. The results show that social networks are crucial in making sense of the experience of recognizing a mental health problem and offering help. Therefore, mental health literacy should be delivered to older people and their social networks, especially GPs, as they could facilitate making sense of mental health-related experiences. In these cases, it is not just relevant to promote knowledge about mental health but also about ageing, having the skills to help understand mental health-related experiences. At the same time, promoting mental health literacy among older people could contribute to empowering them, improving their ability to navigate the system and demanding appropriate help and support.

Additionally, the efforts to reduce stigma should continue, as older people mentioned feeling that what has been done already impacted them being more open to discussing mental health. Talking about mental health through television seems to be an effective way to raise mental health awareness for older people. Public spaces relevant to older people, such as the church, should also be facilitated to discuss mental health, as the sense of common humanity can also contribute to decreasing stigma (Wong et al., 2019).

The experiences of these participants from Southampton reflect that there are still challenges in delivering a holistic service that is articulated with the different services to respond to older people’s needs. Berard et al. (2020) mention that integrating mental health professionals into primary care could reduce older people's time bouncing through the system. Considering the relevance of GPs in access to MHS, this articulation could be fruitful in increasing access rates. Moreover, it is essential not only to articulate needs but to do it within a gerontological perspective, as ageing involves changes that are crucial to consider (Biering, 2019).

## Limitations and future research

Using talking tools had limitations in understanding pathways to treatment as these were only sometimes clear, direct, and sequential. This means that relying only on talking methods can be difficult to explore the transitions between the different pathways. Considering this, creative methods in personal history and time could have helped to explore memories about the different pathways (Edwards & Holland, 2013; Kara, 2015). In this sense, future research could use timelining as a graphic elicitation method to explore life experiences, considering how the interpretations from the past shape the present experiences. This could contribute to a comprehensive and multi-textual representation of participants’ lives, allowing “both participants and researchers to focus on specific aspects of the data to deepen and enrich storytelling” (Sheridan et al., 2011, p.558). Similarly, the life grid method can be used, as this tool can facilitate the recall of detailed data considering the life-course (Parry et al., 1999).

As noted in the Methods section, the main challenge of this study was recruitment. For recruiting participants, different organizations were reached, distributing the flyer among their beneficiaries. Social, community and religious organizations that worked with older people responded, but no mental health organization responded to help distribute the flyer among their contacts. This could indicate that mental health organizations are too busy responding to people’s mental health needs. Nevertheless, it is important to include these organizations as they could be crucial in inviting older people who have used these services. Also, having fewer participants than expected could reflect public stigma associated with mental health, as this could be a sensitive topic where people are not comfortable disclosing their experiences (Braun & Clarke, 2013). Additionally, when recruiting older people with these characteristics, it is important to consider time, as they may need more time to get to know the researcher before considering participating in an interview. For this, it may be important to consider engaging with the older people from the community before starting the recruitment. Given these situations, recruitment represents a challenge for using qualitative methods to understand experiences in access to MHS in older people. However, given the strengths of using these methods, it is important to build a more robust strategy that considers the involvement of mental health organizations, possible public stigma, and time in future studies.

Having four participants was sufficient to explore patterns within the data (Braun & Clarke, 2021), and provided enough insights to answer the research question (Baker & Edwards, 2012). At the same time, these interviews allowed the researcher to represent the diversity of trajectories in older people, considering the richness and complexity of the different experiences (Edwards & Holland, 2013). However, given the heterogeneity of participants and the multiple possibilities of pathways to care, having more interviews could be relevant to exploring further ideas, especially the social component of access to MHS. Moreover, it may be relevant to differentiate access between people who experience common and severe mental health problems as they could have distinctive barriers and facilitators. Also, to include participants that are older people from other ethnic backgrounds, as this study was conducted only with White British people. Furthermore, this study did not include older people with mental health needs who have not accessed mental health services. This may be relevant for understanding other barriers, as there is a high percentage of older people who are experiencing mental health problems and are not accessing them (Sharland et al., 2023).

Future research may also include as participants the social networks of older people who have used MHS, such as GPs, family members, friends, social organizations, and religious organizations. This could contribute to a systemic understanding of how these respond to older people’s mental health needs. Moreover, it could contribute to understanding social meanings attributed to older people’s mental health and the role of these groups in access to mental health. Understanding these discourses and roles could help understand the public dimension of access to MHS in older people, which could be interesting as Mackenzie and Pankratz (2022) agree on the possibility that perceived need can be increased by promoting social engagement and social inclusion.

## Ethical approval and participation consent

This research has ethical approval from the University of Southampton’s Research Integrity and Governance Team (ERGO II) with the project number 81780. A consent form was obtained from all participants.

## Data Availability

Data are available from the first author upon ethical approval.

## References

[cit0001] Baker, S. E., & Edwards, R. (2012). *How many qualitative interviews is enough? Expert voices and early career reflections on sampling and cases in qualitative research* (Research Paper), 1–42. National Centre for Research Methods. https://eprints.ncrm.ac.uk/id/eprint/2273/4/how_many_interviews.pdf

[cit0002] Bao, L., Li, W.-T., & Zhong, B.-L. (2021). Feelings of loneliness and mental health needs and services utilization among Chinese residents during the COVID-19 epidemic. *Globalization and Health*, *17*(1), 1–8. 10.1186/s12992-021-00704-5 33902638 PMC8072077

[cit0003] Beatie, B. E., Mackenzie, C. S., Thompson, G., Koven, L., Eschenwecker, T., & Walker, J. R. (2022). Exploring older adults' experiences seeking psychological services using the network episode model. *Ageing Society*, *42*(1), 48–71. 10.1017/s0144686x20000719

[cit0004] Bengtson, V. L., Burgess, E. O., & Parrott, T. M. (1997). Theory, explanation, and a third generation of theoretical development in social gerontology. *The Journals of Gerontology Series B: Psychological Sciences and Social Sciences*, *52*(2), S72–S88. 10.1093/geronb/52B.2.S72 9060987

[cit0005] Bengtson, V. L., Elder, G. H., & Putney, N. M. (2005). The Lifecourse Perspective on Ageing: Linked Lives, Timing and History. In ML Johnson (Ed.), *The Cambridge handbook of age and ageing* (pp. 493–501). Cambridge University Press. 10.1017/CBO9780511610714.053

[cit0006] Berard, L. D. H., Mackenzie, C. S., Reynolds, K. A., Thompson, G., Koven, L., & Beatie, B. (2020). Choice, coercion, and/or muddling through: Older adults' experiences in seeking psychological treatment. *Social Science Medicine*, *255*, 113011. 10.1016/j.socscimed.2020.113011 32387873

[cit0007] Biering, P. (2019). Helpful approaches to older people experiencing mental health problems: A critical review of models of mental health care. *European Journal of Ageing*, *16*, 215–225. 10.1007/s10433-018-0490-3 31139035 PMC6509324

[cit0008] Bjørlykhaug, K. I., Karlsson, B., Hesook, S. K., & Kleppe, L. C. (2022). Social support and recovery from mental health problems: A scoping review. *Nordic Social Work Research*, *12*(5), 666–697. 10.1080/2156857X.2020.1868553

[cit0009] Braun, V., & Clarke, V. (2013). *Successful qualitative research: a practical guide for beginners*. SAGE Publications Ltd.

[cit0010] Braun, V., & Clarke, V. (2019). Reflecting on reflexive thematic analysis. *Qualitative Research in Sport, Exercise and Health*, *11*(4), 589–597. 10.1080/2159676X.2019.1628806

[cit0011] Braun, V., & Clarke, V. (2021). Can I use TA? Should I use TA? Should I not use TA? Comparing reflexive thematic analysis and other pattern-based qualitative analytic approaches. *Counselling and Psychotherapy Research*, *21*(1), 37–47. 10.1002/capr.12360

[cit0012] Braun, V., & Clarke, V. (2022). *Thematic analysis: A practical guide*. SAGE.

[cit0013] Bretherton, S. J. (2022). The influence of social support, help-seeking attitudes and help-seeking intentions on older Australians' use of mental health services for depression and anxiety symptoms. *International Journal of Aging Human Development*, *95*(3), 308–325. 10.1177/00914150211050882 34747228

[cit0014] Cairney, J., Corna, L. M., & Streiner, D. L. (2010). Mental health care use in later life: Results from a National Survey of Canadians. *Canadian Journal of Psychiatry-Revue Canadienne De Psychiatrie*, *55*(3), 157–164. 10.1177/070674371005500307 20370966

[cit0015] Clark, T., Foster, L., Sloan, L., & Bryman, A. (2021). *Bryman's social research methods* (6th ed.). Oxford University Press.

[cit0016] Dannefer, D., & Phillipson, C. (2013). The study of the life course: Implications for Social Gerontology. In D. Dannefer, & C. Phillipson (Eds.), *The SAGE handbook of social gerontology*. SAGE.

[cit0017] Edwards, R., & Holland, J. (2013). *What is qualitative interviewing*. Bloomsbury Publishing.

[cit0018] Flick, U. (2023). *An introduction to qualitative research* (7th ed.). SAGE.

[cit0019] Forbes, M. K., Crome, E., Sunderland, M., & Wuthrich, V. M. (2017). Perceived needs for mental health care and barriers to treatment across age groups. *Aging Mental Health*, *21*(10), 1072–1078. 10.1080/13607863.2016.1193121 27261055

[cit0020] Garrido, M. M., Kane, R. L., Kaas, M., & Kane, R. A. (2009). Perceived need for mental health care among community-dwelling older adults. *Journals of Gerontology Series B-Psychological Sciences and Social Sciences*, *64*(6), 704–712. 10.1093/geronb/gbp073 19820231 PMC2763014

[cit0021] Garrido, M. M., Kane, R. L., Kaas, M., & Kane, R. A. (2011). Use of mental health care by community-dwelling older adults. *Journal of the American Geriatrics Society*, *59*(1), 50–56. 10.1111/j.1532-5415.2010.03220.x 21198461 PMC3050003

[cit0022] Hammersley, M. (2013). Methodological philosophies, *What is qualitative research*. 21–46. Bloomsbury Academic. 10.5040/9781849666084.ch-002

[cit0023] Hannaford, S., Shaw, R., & Walker, R. (2019). Older adults' perceptions of psychotherapy: What Is It and Who Is Responsible?. *Australian Psychologist*, *54*(1), 37–45. 10.1111/ap.12360

[cit0024] Jorm, A. F. (2000). Mental health literacy: Public knowledge and beliefs about mental disorders. *The British Journal of Psychiatry*, *177*(5), 396–401. 10.1192/bjp.177.5.396 11059991

[cit0025] Kara, H. (2015). *Creative research methods in the Social Sciences: A practical guide*. Policy Press.

[cit0026] Knight, B. G., & Winterbotham, S. (2020). Rural and urban older adults' perceptions of mental health services accessibility. *Aging Mental Health*, *24*(6), 978–984. 10.1080/13607863.2019.1576159 30761911

[cit0027] Lavingia, R., Jones, K., & Asghar-Ali, A. A. (2020). A systematic review of barriers faced by older adults in seeking and accessing mental health care. *Journal of Psychiatric Practice*, *26*(5), 367–382. 10.1097/pra.0000000000000491 32936584

[cit0028] Lumsden, K. (2012). ‘You are what you research’: Researcher partisanship and the sociology of the ‘underdog’. Qualitative Research, 13(1), 3–18.

[cit0029] Mackenzie, C. S., & Pankratz, L. (2022). Perceived need, mental health literacy, neuroticism and self- stigma predict mental health service use among older adults. *Clinical Gerontologist*, *48*, 1–14. 10.1080/07317115.2022.2058440 35400301

[cit0030] Mackenzie, C.S., Pagura, J., & Sareen, J. (2010). Correlates of perceived need for and use of mental health services by older adults in the collaborative psychiatric epidemiology surveys. American Journal of Geriatric Psychiatry, 8(12), 1103–1115. 10.1097/JGP.0b013e3181dd1c06 PMC2992082 20808105

[cit0031] McCormack, B., & Skatvedt, A. (2016). Older people and their care partners' experiences of living with mental health needs: a focus on collaboration and cooperation. *Journal of Clinical Nursing*, *26*, 103–114. 10.1111/jocn.13381 27611624

[cit0032] Muir-Cochrane, E., O'Kane, D., Barkway, P., Oster, C., & Fuller, J. (2014). Service provision for older people with mental health problems in a rural area of Australia. *Aging Mental Health*, *18*(6), 759–766. 10.1080/13607863.2013.878307 24499436

[cit0033] National Health Service. (2015). Psychological Therapies, Annual Report on the use of IAPT services - England, 2014-15. Retrieved 30 June 2023. https://digital.nhs.uk/data-and-information/publications/statistical/psychological-therapies-annual-reports-on-the-use-of-iapt-services/annual-report-2014-15

[cit0034] National Health Service. (n.d.). Older people’s mental health. https://www.england.nhs.uk/mental-health/adults/older-people/

[cit0035] O’Donnell, J., Pybis, J., & Bacon, J. (2021). Counselling in the third sector: to what extent are older adults accessing these services and how complete are the data third sector services collect measuring client psychological distress?. *Counselling and Psychotherapy Research*, *21*(2), 382–392. 10.1002/capr.12393

[cit0036] Parry, O., Thompson, C., & Fowkes, G. (1999). Life course data collection: Qualitative interviewing using the life grid. Sociological Research Online, 4(2), 102–112. 10.5153/sro.233

[cit0037] Pepin, R., Segal, D. L., & Coolidge, F. L. (2009). Intrinsic and extrinsic barriers to mental health care among community-dwelling younger and older adults. *Aging Mental Health*, *13*(5), 769–777. 10.1080/13607860902918231 19882416

[cit0038] Pescosolido, B. (2011). Social Network Influence in Mental Health and Illness, Service Use and Settings, and Treatment Outcomes. In Pilgrim, D., Rogers, A., Pescosolido, B. (Eds.), *The SAGE handbook of mental health and illness*. 512–533. SAGE Publications. 10.4135/9781446200988

[cit0039] Pescosolido, B. A., & Boyer, C. A. (2009). Understanding the Context and Dynamic Social Processes of Mental Health Treatment. In In TL Scheid, & T. N. Brown (Eds.), *A handbook for the study of mental health* (pp. 420–438, 2nd ed.). Cambridge University Press. 10.1017/cbo9780511984945.026

[cit0040] Pettit, S., Qureshi, A., Lee, W., Stirzaker, A., Gibson, A., Henley, W., & Byng, R. (2017). Variation in referral and access to new psychological therapy services by age: an empirical quantitative study. *British Journal of General Practice*, *67*(660), e453–e459. 10.3399/bjgp17X691361 PMC5565877 28583944

[cit0041] Rens, E., Dom, G., Remmen, R., Michielsen, J., & Van den Broeck, K. (2020). Unmet mental health needs in the general population: perspectives of Belgian health and social care professionals. *International Journal for Equity in Health*, *19*(1), 1–10. 10.1186/s12939-020-01287-0 PMC7526210 32993667

[cit0042] Reynolds, K. A., Mackenzie, C. S., Medved, M., Dudok, S., & Koven, L. (2022). Older adults' mental health information preferences: a call for more balanced information to empower older adults' mental health help-seeking. *Ageing Society*, *43*, 1–30. 10.1017/s0144686x21001896

[cit0043] Sharland, E., Rzepnicka, K., Schneider, D., Finning, K., Pawelek, P., Saunders, R., & Nafilyan, V. (2023). Socio-demographic differences in access to psychological treatment services: Evidence from a national cohort study. *Psychological Medicine*, *53*, 1–12. 10.1017/S0033291723001010 PMC10721408 37194490

[cit0044] Sheridan, J., Chamberlain, K., & Dupuis, A. (2011). Timelining: visualizing experience. *Qualitative Research*, *11*(5), 552–569. 10.1177/1468794111413235

[cit0045] Simões de Almeida, R., Trigueiro, M. J., Portugal, P., de Sousa, S., Simões-Silva, V., Campos, F., Silva, M., & Marques, A. (2023). Mental health literacy and stigma in a Municipality in the North of Portugal: A cross-sectional study. *International Journal of Environmental Research and Public Health*, *20*(4), 3318. 10.3390/ijerph20043318 36834014 PMC9962300

[cit0046] Thoits, P. A. (2011). Perceived social support and the voluntary, mixed, or pressured use of mental health services. *Society and Mental Health*, *1*(1), 4–19. 10.1177/2156869310392793

[cit0047] Volkert, J., Andreas, S., Harter, M., Dehoust, M. C., Sehner, S., Suling, A., Ausin, B., Canuto, A., Crawford, M. J., Da Ronch, C., Grassi, L., Hershkovitz, Y., Munoz, M., Quirk, A., Rotenstein, O., Santos-Olmo, A. B., Shalev, A. Y., Strehle, J., Weber, K., Schulz, H., Härter, M., Ausín, B., Muñoz, M., Wegscheider, K., & Wittchen, H. (2018). Predisposing, enabling, and need factors of service utilization in the elderly with mental health problems. *International Psychogeriatrics*, *30*(7), 1027–1037. 10.1017/s1041610217002526 29198254

[cit0048] Walters, K., Falcaro, M., Freemantle, N., King, M., & Ben-Shlomo, Y. (2018). Sociodemographic inequalities in the management of depression in adults aged 55 and over: an analysis of English primary care data. *Psychological Medicine*, *48*(9), 1504–1513. 10.1017/S0033291717003014 29017624

[cit0049] Wong, C. C. Y., Knee, C. R., Neighbors, C., & Zvolensky, M. J. (2019). Hacking stigma by loving yourself: A mediated-moderation model of self-compassion and stigma. *Mindfulness*, *10*(3), 415–433. 10.1007/s12671-018-0984-2

